# Proton Therapy for Bilateral Breast Cancer Maximizes Normal-Tissue Sparing

**DOI:** 10.14338/IJPT-22-00041.1

**Published:** 2023-03-06

**Authors:** Eric D. Brooks, Raymond B. Mailhot Vega, Emma Vivers, Teena Burchianti, Xiaoying Liang, Lisa R. Spiguel, Bharti Jasra, Nancy P. Mendenhall, Oluwadamilola T. Oladeru, Julie A. Bradley

**Affiliations:** 1 Department of Radiation Oncology, University of Florida College of Medicine, Gainesville and Jacksonville, FL, USA; 2 University of Florida Health Proton Therapy Institute, Jacksonville, FL, USA; 3 Department of Radiation Oncology, Mayo Clinic, Jacksonville, FL, USA; 4 Department of Surgery, University of Florida College of Medicine, Gainesville, FL, USA; 5 Department of Surgery, University of Florida Jacksonville College of Medicine, Jacksonville, FL, USA

**Keywords:** particle therapy, radiation therapy, dosimetric analysis, oncology outcomes

## Abstract

**Purpose:**

Treatment for bilateral breast cancer with radiation therapy is technically challenging. We evaluated the clinical and dosimetric outcomes of a small series of patients with synchronous bilateral breast cancer, including a photon dosimetric comparison, to identify optimal treatment planning approaches.

**Materials and Methods:**

We reviewed a registry of patients (simultaneously) diagnosed with synchronous bilateral breast cancers who underwent postoperative definitive adjuvant proton therapy at our institution between 2012 and 2021. All patients were treated with double-scattered proton or pencil-beam scanning therapies. For comparison, intensity-modulated radiation therapy photon plans optimized for organ sparing and coverage were generated after treatment.

**Results:**

Six patients were included. The median patient age was 66 years; all were female with no history of breast cancer or radiation therapy. Two (33%) patients received breast/chest wall–only treatments, 1 (17%) required breast plus level I axillary treatment to one side and breast plus regional nodal irradiation (RNI) to the other, and 3 (50%) received bilateral breast/chest plus RNI; dosimetric results are reported for each group's median. Analysis showed clinical target coverage was comparable between proton and photon techniques (V95% of 96.4% with proton, 97.8% with photon). However, protons could deliver superior organ sparing at clinically relevant dose metrics for virtually all structures: a 6.7 Gy absolute reduction in the mean heart dose (7.5 Gy with photons to 0.7 Gy with protons), a 47% to 57% relative reduction in D_0.1cm3_ to coronary arteries, a 54% relative reduction in lung V20 Gy, and an absolute 7.6 Gy reduction to the brachial plexus. There was also greater esophagus and spinal cord sparing. The overall survival rate was 100% at 1.5 years of median follow-up (0.5-4.9), and all patients were free of disease. For toxicity, all patients had some form of acute side effects: 66% experienced grade 2 breast/chest pain or soreness; 100% had grade 2 radiation dermatitis or skin induration; 33% had grade 2 fatigue; and 17% had grade 2 esophagitis (per the Common Terminology Criteria for Adverse Events [CTCAE] version 5.0; US National Cancer Institute, Bethesda, Maryland). Subacute toxicity (within 6 months) was observed for 17% of patients with delayed onset of grade 3 dermatitis in the setting of preexisting lupus, 17% with a delayed surgical wound complication, and 17% with grade 2 soft tissue fibrosis. No grade 4 or 5 events were observed.

**Conclusions:**

Substantial dose reductions to multiple organs at risk while maintaining target coverage make proton the preferred modality for bilateral breast cancer treatment when available.

## Introduction

Primary synchronous bilateral breast cancer rates are increasing. While the incidence has been historically low (between 1% and 4%), the modern use of magnetic resonance imaging (MRI) for both screening and diagnostic workups has led to a 3% to 5% increase in the rate of synchronous breast cancer detection [1–4]. For women with bilateral breast cancer with indications for postmastectomy radiation therapy (PMRT) or radiation as a component of breast conservation, sparing the heart and lungs with a broad field sufficient to cover the entire anterior chest can be daunting. Traditional external beam techniques (eg, 3-dimensional [3D] photon, 3D photon-electron matching, tomotherapy, intensity-modulated radiation therapy [IMRT], volumetric modulated arc therapy [VMAT]) often exceed or approach the limits of important organ tolerances, most notably the heart and lungs [5–13]. This is particularly true when regional nodes are treated, such as the internal mammary chains near the heart and its coronary vessels or the supraclavicular basins adjacent to the lung apices. The hazard associated with traditional photon methods in these scenarios has been recognized [5, 8–13]. Numerous technical reports outline the complexity of treatment delivery for patients with bilateral breast cancer with photon treatment and the trade-offs between target coverage and normal tissue avoidance that are frequently made. However, recent advances in proton therapy have emerged as an elegant solution for treating bilateral breast cancer. Proton therapy enables the treatment of large fields while simultaneously sparing the local critical tissues.

Here we present a dosimetric comparative study of patients with bilateral breast cancer treated at the University of Florida Health Proton Therapy Institute. We aim to evaluate the differences between photon and proton therapy concerning target coverage and organ avoidance in the setting of bilateral breast cancer treatment.

## Materials and Methods

### Patient Identification

Under institutional review board approval and with informed consent, we reviewed a prospective registry of patients with breast cancer diagnosed between 2012 and 2021 at the University of Florida Health Proton Therapy Institute. All patients who underwent bilateral proton therapy for synchronous bilateral breast cancer diagnosis were included. However, patients were excluded if they did not receive radiation, if they only received unilateral radiation, or if they received photon radiation for their bilateral breast cancer.

### Description of Treatment Plan and Delivery

The methods for treating bilateral breast cancer with proton therapy have been previously described [14, 15]. All patients were simulated on a breast board with an incline of 5° to 15°, with the head neutral and the arms over the head. An open-faced mask was sometimes used if regional nodal irradiation (RNI) was delivered (2 of 4 patients); the face mask is now routinely used in RNI cases at our center. Vacuum bags, or other immobilization equipment, were not routinely used to facilitate easier repositioning during treatment setup. Free breathing was used, as interplay effects are a minor concern when the breathing motion is in the direction of beam delivery [16, 17].

Contours for all plans (double-scattered [DS] or pencil-beam scanning [PBS]) were done per RTOG atlas guidelines (when applicable and based on the date treated), incorporating the RadComp Breast Atlas guidelines. RNI included the axillary levels I-III, the first 3 intercostal spaces of the internal mammary chain, and the supraclavicular nodes (SCV). Patients receiving postmastectomy radiation or whole-breast radiation therapy (WBRT) plus RNI had these RNI levels included for treatment (axillae I-III/SCV/internal mammary node [IMN]). Furthermore, at our center, the breast clinical target volume (CTV) is subtracted 5 mm from the skin, and the chest wall CTV is subtracted 3 mm from the skin for skin sparing.

Passive DS proton plans were generated using Eclipse (Eclipse Treatment Planning System, version 11.0.47, Varian Medical Systems, Crawley, United Kingdom). Two matchline changes were used to reduce inhomogeneity at field junctions. Proton PBS plans were robustly planned and optimized on RayStation versions 6 and 8A (RaySearch Laboratories, Stockholm, Sweden) using a Monte Carlo dose engine [15]. All plans used 2 to 4 fields to account for target geometry, delivery technique, and field size. Predominately en face beams were used perpendicular to lung motion to reduce uncertainty owing to respiratory motion. Single-field optimization (SFO) was used to improve robustness whenever possible for PBS plans. For large field sizes and complex anatomies, multifield optimization (MFO) was used for each bilateral side, with multiple beams per side to reduce delivery uncertainty. PBS plans were robustly optimized on CTV structures with 5-mm setup uncertainty and 3.5% range uncertainty for independent beams. During treatment, verification computed tomography (VFCT) scans were obtained at weeks 2 and 4 to assess for anatomy changes that would require replanning. Replanning was prompted when clinical goals were exceeded compared with the original plan after an imaging examination confirmed that dose differences were the result of tissue changes during treatment. The prescriptions used at our center generally include 42.4 Gy in 16 fractions for WBRT with 7.95 Gy to 10.6 Gy delivered in a 3- to 4-fraction tumor bed boost, or 50 Gy in 25 fractions for comprehensive chest wall or breast plus RNI radiation therapy (ie, PMRT or WBRT + RNI) with 10 Gy up to 20 Gy in 5- to 10-fraction scar/tumor bed boosts depending on margin status and the presence of undissected or residual nodes.

All proton therapy treatments were delivered on consecutive weekdays. Image-guided radiation therapy consisted of daily kilovolt imaging. In addition, VFCT scans were routinely obtained every 2 weeks during treatment to detect tissue changes possibly requiring treatment modifications.

IMRT photon plans were then generated after treatment. Free-breathing averaged 4-dimensional (4D) CT scans were used for both the proton and photon plans for ease of comparison. The IMRT plans were created using RayStation version 8A (RaySearch) with static multileaf collimation delivery. These plans were rigorously optimized to achieve optimal coverage and organ tissue sparing with 12 static fields. Notably, the optimization objectives for both the proton and IMRT plans were as follows: the targets were the first priority, with the objective of 98% of the dose covering 98% of the individual CTV, and 95% of the dose covering 95% of the combined planning target volumes. The maximum dose goal was <110% for the IMRT and <104% for the proton plans. The second-priority objectives were the total lung goals of V20 Gy <30% and V5 Gy <65%, the heart mean <5 Gy, and a maximum dose of 50 Gy to the esophagus. The lower-priority objectives were brachial plexus maximum dose <60 Gy, spinal cord <45 Gy, left anterior descending coronary artery (LAD) 0.1 cm^3^ <3 Gy, and right coronary artery (RCA) 0.1 cm^3^ <3 Gy. IMRT was selected as the comparative modality given its many superior attributes, as demonstrated in previously cited photon comparison studies for bilateral breast cancer treatment, making it a preferred comparative technique to proton therapy in this setting [5, 18].

### Statistics and Analysis

Simple descriptive statistics were used to compare the IMRT photon and proton plans for the initial phase of treatment (excluding the boost phases). Given the small sample size, the median was used to represent the average for any statistics. Comparative statistics, including the use of significance, were precluded owing to the small sample size. Dosimetric comparisons were made using ProKnow DS software (Elekta Solutions AB, Stockholm, Sweden). Dosimetric data were exported to Excel (Microsoft, Redmond, Washington) for further numerical analysis. All numerical data were analyzed in SPSS version 21.0 (IBM Corporation, Armonk, New York).

## Results

### Patient Characteristics

We identified 6 patients with synchronous bilateral breast cancers, diagnosed simultaneously, who underwent adjuvant proton therapy at the University of Florida Health Proton Therapy Institute between 2016 and 2020. Patient and tumor characteristics are summarized in **Table 1**. The American Joint Committee on Cancer (AJCC) 8th edition anatomic staging was applied to all patients [19]. The median age of patients was 65 years, all were female, and none had a history of breast cancer or radiation therapy.

**Table 1.** Patient, treatment, and tumor characteristics for 6 women treated with proton therapy for bilateral breast cancer.

**Patient**	**Age, y**	**Prescription dose, Gy (left; right)**	**Target**	**Tumor characteristics**
1	75	42.4; 50.0	Intact breast	Stage IA pTcN0(sn)(i+), G3, IDC, ER+/PR+/HER2-
				Stage IIA pT1bN1a(sn)M0, G1, IDC and ILC, ER+/PR+/HER2-
2	62	46.0; 46.0	Intact breast	Stage IA pT1cN0M0, G1, IDC, ER+/PR+/HER2-
				Stage IA pT1cN0M0, G2, ILC, ER+/PR+/HER2-
3	68	50.0; 50.0	Intact breast	Stage IIA ypT1bN1M0, G3, IDC, ER+/PR+HER2-
				Stage IIA ypT1bN1M0, G2, IDC, ER+/PR+/HER2-
4	64	50.0; 50.0	Intact breast	Stage 0 pTisN0M0, G2, DCIS, ER+
				Stage IA pT1cN0M0, G2, ILC, ER+/PR+/HER2-
5	49	50.0; 50.0	Chest wall	Stage IIB pT3N0M0, G3, IDC, TNBC
				Stage IIB pT2N1M0, G3, IDC, TNBC
6	70	50.0; 50.0	Intact breast	Stage IIA pT2N2M0, G2, IDC, ER+/PR+/HER2-
				Stage IA pT1aN1M0, G3, IDC, ER+/PR+/HER2 unknown

**Abbreviations:** G, tumor grade; IDC, invasive ductal carcinoma; ER, estrogen receptor; PR, progesterone receptor; HER2, receptor tyrosine-kinase erbB-2; ILC, invasive lobular carcinoma; TNBC, triple negative breast cancer.

Regarding tumor characteristics, the clinical T stage for all tumors comprised 67% (8/12) stage T1; 17% (2/12), T2; 8% (1/12), T3-4; and 8% (1/12), Tis DCIS (ductal carcinoma in situ). A total of 67% (4/6) of the patients had at least 1 node positive for tumor, 83% (5/6) had at least 1 estrogen receptor positive tumor; 0% (0/6) had Her-2 positive tumors, and 33% (2/6) had tumor profiles or histologies that differed between the bilateral sides.

Among the 6 patients, 10 (83%) breasts underwent lumpectomy as treatment, 2 (17%) underwent a mastectomy, 10 (83%) underwent sentinel lymph node biopsy, and 1 (8%) underwent axillary lymph node dissection. No axillary staging was done for the breast with DCIS histology. No patients had an expander or implant reconstruction prior to proton therapy treatment. In addition, 50% (3/6) received chemotherapy (adjuvant or neoadjuvant). The chemotherapy regimens were either dose-dense adriamycin, cytoxan, and taxol (ddAC/T) used in 2 (67%) of the patients receiving chemotherapy or taxotere and cytoxan (TC) used in 1 (33%).

For radiation therapy, 2 (33%) patients required breast/chest-only treatment on both sides, 1 (17%) required breast plus level I axillary treatment on one side and breast plus RNI treatment on the other, and 3 (50%) patients required breast/chest plus RNI bilaterally (**Table 2**). All patients were treated with proton-passive DS (n = 2) or PBS (n = 4) therapies. All patients successfully completed their proton course of treatment. Two of the 6 patients (33%) required replanning as a result of tissue changes during treatment. In addition, 2 patients had minor breaks (1- to 4-day periods) because of moist desquamation dermatitis.

**Table 2.** Proton therapy treatment technique for all 6 women included in the analysis.

**Patient**	**Parameter**	**Left breast treatment**	**Right breast treatment**
1	Target	WBRT + low axillae	WBRT + RNI
	Dose	42.4 Gy in 16	50.0 Gy in 25
	Boost	Tumor bed, 10.6 Gy in 4	Tumor bed, 10.0 Gy in 5
	Technique	PBS, 2 fields, SFO	PBS, 2 fields, SFO
2	Target	WBRT	WBRT
	Dose	46.0 Gy in 23	46.0 Gy in 23
	Boost	Tumor bed, 14.0 Gy in 7	Tumor bed, 14.0 Gy in 7
	Technique	PBS, 2 fields, SFO, alternating 1 field/d at full dose	PBS, 2 fields, SFO, alternating 1 field/d at full dose
3	Target	WBRT + RNI	WBRT + RNI
	Dose	50.0 Gy in 25	50.0 Gy in 25
	Boost	None	None
	Technique	PBS, 2 fields, SFO, alternating 1 field/d at full dose	PBS, 2 fields, SFO, alternating 1 field/d at full dose
4	Target	WBRT	WBRT
	Dose	50.0 Gy in 25	50.0 Gy in 25
	Boost	None	10.0 Gy in 5 (DS, 1 field)
	Technique	PBS, 2 fields, SFO, alternating 1 field/d at full dose	PBS, 2 fields, SFO, alternating 1 field/d at full dose
5	Target	PMRT^a^	PMRT
	Dose	50.0 Gy in 25	50.0 Gy in 25
	Boost	None	None
	Technique	PBS, 2 fields, SFO, alternating 1 field/d at full dose	PBS, 2 fields, SFO, alternating 1 field/d at full dose
6	Target	WBRT + RNI	WBRT + RNI
	Dose	50.0 Gy in 25	50.0 Gy in 25
	Boost	Tumor bed, 10.0 Gy in 5; IMNs, 16.0 Gy in 8^b^	None
	Technique	DS, 4 fields, alternating 2 fields/d at full dose	DS, 4 fields, alternating 2 fields/d at full dose

**Abbreviations:** WBRT, whole-breast radiation therapy; RNI, regional node irradiation; PBS, proton pencil-beam scanning; SFO, single-field optimization; DS, proton double scatter; PMRT, postmastectomy radiation therapy; IMN, internal mammary nodes.

a PMRT includes ipsilateral chest wall, axillary levels I-III, supraclavicular nodes, and IMN.

b Both boosts were DS, 2 fields.

The median follow-up for all 6 patients was 1.5 years (0.5-4.9 years) starting from the end of proton therapy. Local control, regional control, and distant control rates were 100%; no patient experienced any recurrence or metastasis. The overall survival rate was 100% at 1.5 years. Acute grade 2+ transient toxicity (per the Common Terminology Criteria for Adverse Events [CTCAE], version 5.0; US National Cancer Institute, Bethesda, Maryland) was seen during treatment in all patients. A total of 66% (4 of 6) of patients had grade 2 breast/chest pain or soreness, 100% (6 or 6) had grade 2 radiation dermatitis or skin induration, 33% (2 or 6) had grade 2 fatigue, and 17% (1 or 6) had grade 2 esophagitis. Subacute toxicity (within 6 months) was observed for 17% (1 or 6) of patients with delayed onset of grade 3 dermatitis in the setting of preexisting lupus, 17% (1 or 6) with a delayed surgical wound complication, and 1 (17%) with grade 2 soft tissue fibrosis. No grade 4 or 5 events were observed.

### Target Coverage

Target coverage was assessed for the breast and/or chest wall CTV in addition to the axillae, supraclavicular, and internal mammary nodal CTVs (when included), as originally contoured. Analysis showed target coverage was comparable between proton and photon techniques (median CTV coverage V95% of 96.4% with protons versus 97.8% with photons; median CTV D95% of 95.8% with protons versus 97.1% with photons) (**Table 3**).

**Table 3.** Patient-matched proton and photon dosimetric comparison plans for all 6 women with bilateral breast cancer included in the analysis.

**Result**	**Proton, median (range)**	**Photon, median (range)**
Heart		
D_mean_ (Gy)	0.7 (0.0–2.6)	7.4 (4.9–7.9)
V25 Gy (%)	0.1 (0.0–3.4)	0.7 (0.0–3.6)
V10 Gy (%)	2.2 (0.0–7.9)	9.2 (0.7–16.0)
V5 Gy (%)	3.7 (0.1–11.4)	83.0 (29.7–100.0)
V5 Gy (cm^3^)	26.8 (0.6–68.3)	550.4 (180.0–625.4)
LAD		
D_mean_ (Gy)	1.7 (0.4–7.5)	11.5 (6.6–14.4)
D_0.1cm3_ (Gy)	9.5 (1.6–17.9)	18.0 (11.3–39.2)
RCA		
D_mean_ (Gy)	0.6 (0.0–5.3)	6.5 (5.0–17.5)
D_0.1cm3_ (Gy)	3.2 (0.0–22.7)	7.4 (6.4–28.3)
Ventricles		
V25 Gy (%)	0.09 (0.0–0.9)	0.05 (0.0–4.2)
V5 Gy (%)	2.4 (0.0–6.8)	78.8 (34.2–100.0)
Lungs (total)		
V20 Gy (%)	14.1 (0.1–26.3)	31.3 (13.8–34.6)
V10 Gy (%)	23.3 (0.2–44.7)	76.9 (35.6–93.2)
V5 Gy (%)	30.3 (0.5–58.4)	96.8 (59.8–100.0)
Brachial plexus, left		
D_0.1cm3_ (Gy)	45.3 (0.1–52.4)	51.8 (22.5–58.3)
Brachial plexus, right		
D_0.1cm3_ (Gy)	47.8 (0.0–49.5)	56.5 (19.5–59.04)
Esophagus		
D_mean_ (Gy)	4.4 (0.0–12.9)	13.8 (3.7–19.5)
D_max_ (Gy)	41.6 (0.0–50.0)	49.1 (7.1–56.7)
Liver		
D_700cm3_ (%)	0.01 (0.0–0.1)	8.1 (2.7–16.0)
Thyroid		
D_mean_ (Gy)	24.5 (0.0–47.4)	41.2 (0.8–50.8)

**Abbreviations:** LAD, left anterior descending coronary artery; RCA, right coronary artery.

### Organ Sparing

**Figure 1.**
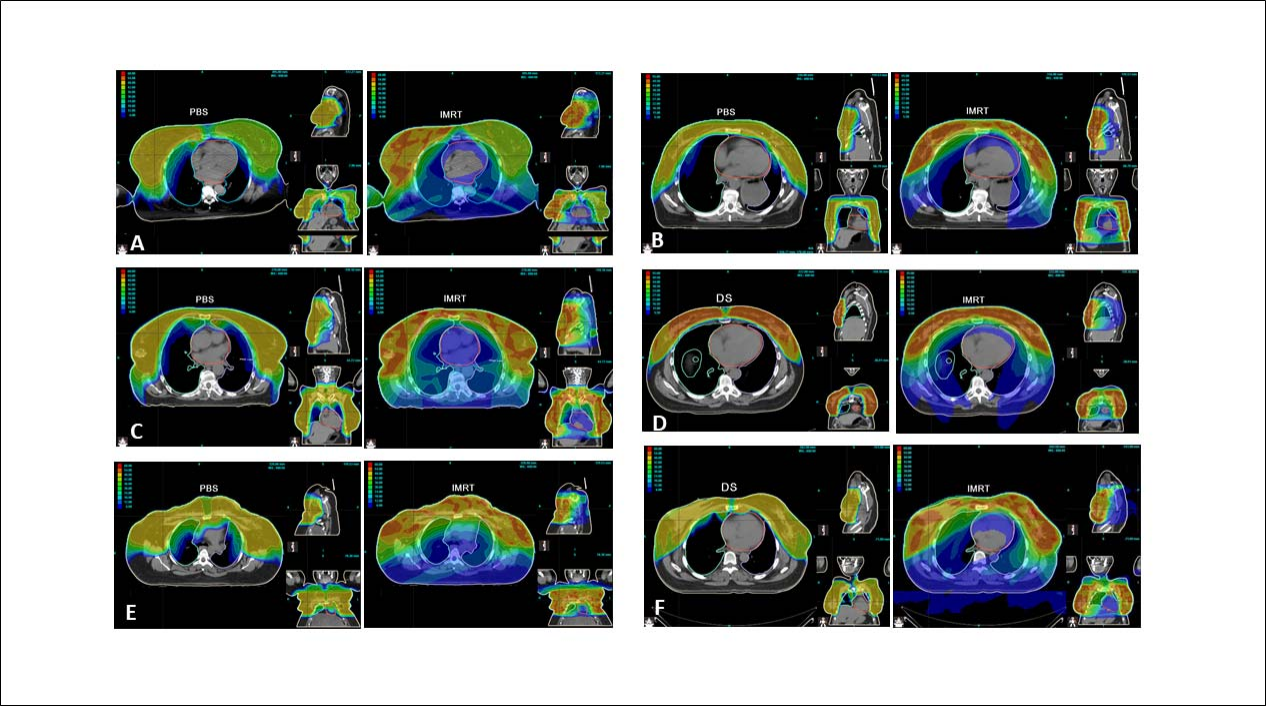
Proton- (left) and photon-based (right) IMRT comparison plans for all 6 patients demonstrate the organ-sparing advantages of proton therapy in bilateral breast cancer treatment (panels A through F represent patients 1 through 6). All Panels show colorwash representations of treatment and include dose-level legends in the top left-hand corner. The high treatment dose is yellow-red. The low-intermediate dose is blue-green. Abbreviation: IMRT, intensity-modulated radiation therapy.

**Figure 2.**
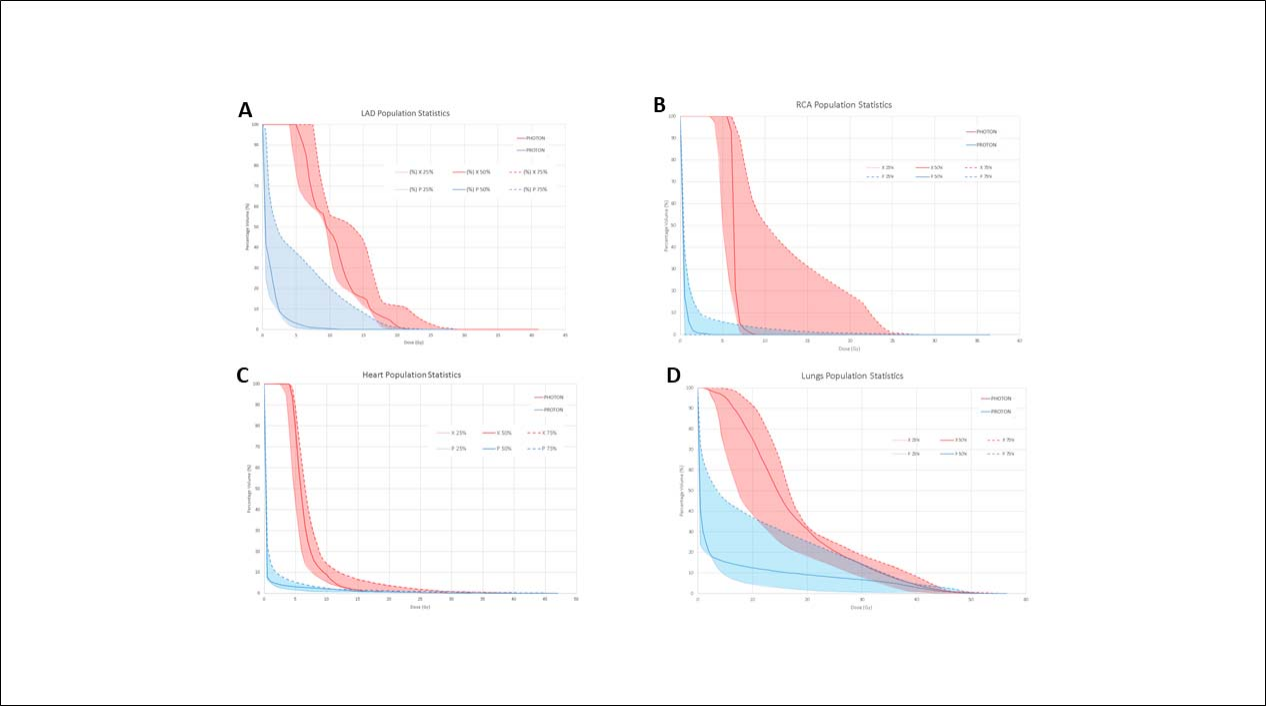
Panels A through D represent the DVH comparisons between the proton- and photon-based IMRT plans (n = 6 for each). The median doses (solid lines) and variance (dashed lines, 25% and 75% quartiles) are shown for both groups. The variance is color-filled for each group: blue represents protons; red represents photons. Substantial tissue-sparing benefits can be observed when using protons compared with photons for the LAD (panel A), RCA (panel B), heart (panel C), and lungs (panel D). Abbreviations: DVH, dose-volume histogram; IMRT, intensity-modulated radiation therapy; LAD, left anterior descending coronary artery; RCA, right coronary artery.

Protons were able to deliver superior organ sparing at clinically relevant dose metrics for virtually all structures (**Figure 1**). This was achieved for the heart, LAD, RCA, ventricles, lungs, brachial plexuses, esophagus, liver, and spinal cord (**Table 3**, **Figure 2**).

Notably, there was a median 10-fold reduction and absolute 6.7-Gy reduction in mean heart dose with protons compared with photons (median mean heart dose 7.5 Gy with photons to 0.7 Gy with protons). The median heart V25 Gy was similar between protons (0.2%) and photons (0.7%). However, the intermediate median heart V10 Gy was substantially reduced from 9.3% with photons to 2.2% with protons (4.2-fold relative reduction, absolute 7.1% reduction). Additionally, the median V5 Gy to the heart was reduced from 83.3% with photons to 3.7% with protons (22-fold relative reduction, absolute 79.6% reduction).

Protons additionally achieved superior sparing for the coronary vessels. The median LAD D_mean_ was 11.6 Gy with photons compared with 1.7 Gy with protons (6.7-fold relative reduction, absolute 9.9 Gy reduction). Similarly, the RCA average D_mean_ was reduced from 6.6 Gy with photons to 0.6 Gy with protons (10-fold relative reduction, absolute 5.9 Gy reduction). The mean LAD D_0.1cm3_ was also reduced by 46% from 18.1 Gy with photons to 9.6 Gy with protons, mirrored by a 57% relative reduction of the RCA D_0.1cm3_ from 7.4 Gy with photons to 3.2 Gy with protons. Thus, the use of protons represented absolute reductions in median D_0.1cm3_ of 8.5 Gy for the LAD and 4.2 Gy for the RCA. The cardiac ventricles also had substantial sparing with a 32-fold relative reduction in V5 Gy with protons compared with photons.

The median total lung V20 Gy was reduced from 31.3% with photons to 14.2% with protons. In addition, other low- to intermediate-dose lung metrics were reduced with protons compared with photons, including a median V10 Gy reduction from 77.0% with photons to 23.3% with protons and a median V5 Gy reduction from 96.8% with photons to 30.4% with protons.

Regarding the brachial plexus, the median D_0.01cm3_ was reduced by 7.6 Gy with protons compared with photons. The median esophageal D_mean_ was also reduced with protons by 68%, from 13.9 Gy with photons to 4.5 Gy with protons. The median liver D_700cm3_ was reduced from 8.2 Gy with photons to 0.01 Gy with protons. A reduction was also observed in the median thyroid D_mean_ from 41.3 Gy with photons to 24.5 Gy with protons. Lastly, the median spinal cord D_max_ dropped 25.1 Gy, from 32.1 Gy with photons to 7.0 Gy with protons, which may be especially important for patients who later require palliative treatment to the spine owing to metastasis where overlap avoidance is critical.

## Discussion

To date, this is one of the few reports highlighting how proton technology can assist patients with bilateral breast cancers, which are some of the most technically challenging cases. With a median follow-up of 1.5 years (0.5-4.9 years), no in-field recurrences or marginal misses have occurred, supporting accurate targeting and treatment delivery.

Like ours, previous reports have also found that the mean heart dose can be reduced nearly 5-fold (from 9.98 Gy to 2.12 Gy), and lung dose metrics, such as V5 Gy, can be halved (from 97.9% to 39.8%) by using proton rather than photon therapy [14]. Other reports corroborate the superiority of protons over photons in the avoidance of adjacent organs at risk (OARs) when treating patients with bilateral breast cancer [18, 20, 21].

Often, in studies evaluating photon-based treatments for bilateral breast cancers [5–9, 14, 20], the mean heart dose averages approximately 8 Gy, which is a dose far exceeding that advised by major guidelines to sufficiently spare the myocardium (RTOG/Quantitative Analyses of Normal Tissue Effects in the Clinic/National Comprehensive Cancer Network). In addition, this study and other corroborating publications show that protons can substantially reduce the mean heart dose in bilateral breast cancer treatment to <3 Gy, which is generally considered within accepted constraints [14, 18]. Notably, in the largest dosimetric comparative study to date, the mean heart dose was reduced from an average of 7.2 Gy to 0.7 Gy with proton versus photon for bilateral breast cancer patients [21]. This is important because the dose-cardiac risk relationship is well-defined in breast cancer [22] and validated in other disease-site studies [23], suggesting that for each additional 1 Gy mean heart dose, an approximate 7% relative increase in the risk of cardiac injury is conferred. Thus, proton therapy could offer up to a 49% relative reduction in potentially lethal cardiac events compared with photons in patients undergoing bilateral breast cancer treatment.

The cardiac benefit of proton therapy in bilateral breast cancer treatment is not purely indicated by the mean heart dose. Our findings that the D_0.1cm3_ maximum dose to the coronary arteries (LAD and RCA) was almost halved and the D_mean_ reduced 8-fold in bilateral breast cancer cases is indicative of proton therapy's dosimetric superiority over photon therapy in treating such patients. New data continue to emerge, showing that sparing these cardiac substructures may be especially critical in avoiding late cardiac complications [24]. However, this type of conformal avoidance has only been more recently entertained through such techniques as proton therapy or advanced photon planning. Most notably, the importance of recording and limiting the dose to the LAD is promoted and may reduce the chance of radiation-induced stenosis [25, 26]. The coronaries, and their branches, represent small- to medium-sized vessels on which radiation therapy is known to have its most profound effects in terms of atherosclerosis and fibrosing [27, 28]. The branch-point nature of coronaries at the base of the aorta, as well as further down their course, make them especially prone to atherosclerotic progression because of the hemodynamic flow characteristics of vessels at branch points [29, 30]. Therefore, because both the LAD and RCA may receive a significant dose during bilateral breast cancer treatment, a compromise in healthy collateralization over time may not occur where one healthy vessel compensates for the narrowing of another [31]. This makes sparing at least 1 of the coronaries in bilateral treatment (if not both) critically important in preventing risks to the heart over subsequent years. We show that coronary sparing can best be achieved when proton therapy is used for bilateral breast cancer treatment rather than modulated photon therapy. Thus, this further advances the case that proton is heart sparing in this setting. Notably, this coronary sparing has been corroborated in other critical reports [21].

Although cardiac sparing may appear to be the most substantive dosimetric benefit with proton therapy compared with photon therapy in treating patients with bilateral breast cancer, we also found that superior dose falloff and beam shaping conferred by protons, owing to the sharp penumbra, Bragg peak, and spot positioning, can reduce the dose to other adjacent organs.

Reducing the D_max_ to the brachial plexus (which may reduce the overall chance of long-term plexopathy [32–34]), limiting the mean dose, V5 Gy, and V20 Gy to the lungs (which can lessen the chance of pneumonitis and second malignancy [35, 36]), and sparing other organs, such as the esophagus, all contribute to the justification of proton therapy as an advantageous method for patients with bilateral breast cancer. This is especially the case as meta-analyses now show a log-linear relationship with brachial plexopathy such that limiting the dose to the plexus—even within traditional thresholds—may provide a benefit [37].

In addition, it is suggested that using protons instead of photons for ipsilateral treatments may reduce the risk for secondary malignancies by 38% [38]. This advantage may be even more pronounced in bilateral treatments where much more extensive areas are exposed. These results have demonstrated a clear rationale for using proton therapy for bilateral breast cancer irradiation (when available) to maintain coverage goals while offering considerable dose reduction to life-limiting radiosensitive adjacent organs.

Importantly, bilateral treatment is unique in that it can cause damage to complementary structures on bilateral sides, which furthers the rationale for proton therapy in this setting. For example, avoidance of the contralateral lung in ipsilateral treatment cannot be obtained during bilateral treatment. This exposure to the full set of organs in a bilateral plan is of particular importance in consideration of the dose not only to the lungs but also to the brachial plexuses, coronaries, and anterior heart surface(s). Preserving at least 1 hemi-organ structure in the event that the other experiences toxicity is paramount so that the chance for damage and dysfunction to both body side structures, and thereby incurring debilitating toxicity, is minimized (eg, minimizing dose to each plexus as much as possible to mitigate the occurrence of bilateral plexopathy). The reduction of dose to those complementary structures, and therefore disability, seems to be best achieved with proton therapy versus IMRT.

When compared with photon therapy, the Particle Therapy Consensus Group (PTCG) formally recognizes that protons can improve full CTV coverage for comprehensive target coverage in ipsilateral breast treatment while sparing many normal tissues, especially when the IMN chain is required. The PTCG has also published consensus data regarding the evidence for the benefits of proton in breast cancer treatment, including bilateral treatment [39].

While the technical aspects of proton therapy planning and delivery for bilateral breast cancer cases are beyond the scope of this work, such guides have been reported [14, 20]. In these reports, we and others reflect on an array of technical options to treat patients with proton therapy, depending primarily on patient anatomy, reconstruction, the size of the fields required, and the delivery techniques available (see Methods section). Importantly, centers offering proton therapy for bilateral breast cancer should employ expertise in the planning, delivery, and quality assurance phases of treatment to ensure the robustness, safety, and effectiveness of plans. In light of these results and others, we conclude that proton therapy offers substantial advantages over photon therapy in the context of bilateral breast cancer treatment.

This study is not without its limitations. Chief among them is the small sample size that precludes comparative statistics for establishing the statistical significance of differences between proton and photon treatments.

Second, clinical outcome comparisons between modalities are not available because of the rarity of the condition and the time to witness outcome data, such as early cardiac changes on advanced imaging, which can take years to observe. We also acknowledge limited data for intact breast and cosmesis outcomes. Additionally, there is a paucity of data concerning the end-of-range aspects of proton therapy on OARs, which could have very meaningful clinical effects and long-term implications for breast cancer proton therapy survivors, especially in the bilateral treatment setting. These factors make comparative clinical studies in such a small patient population challenging, if not impractical. They also highlight the need for individual and collaborative center results to be published on this small but uniquely important group of patients so that best management practices can be adopted as they evolve and long-term outcomes, applied to guide management as they matriculate.

Third, we include 2 patients here who had breast-only treatment. Given that photon plans for bilateral breast-only treatment (without RNI) can produce excellent plans, the advantages we present may be most applicable to bilateral breast cancer patients who require RNI. This is especially recognized in the context of widespread deep inspiration breath hold (DIBH) adoption, which drops the heart posterior-inferior to the photon treatment fields while pushing the chest away anteriorly to create excellent heart and lung sparing in many bilateral breast-only cases. However, some patients may benefit from proton therapy for bilateral breast-only treatment. Such patients include those whose anatomy is still unfavorable following DIBH (eg, the heart minimally separates, the heart continues to encroach along a material length of the chest wall, the lateral border of breast tissue makes heart or lung sparing encumbered with regard to coverage), those who have underlying conditions whereby even low dose reduction to the heart or lungs could be advantageous (eg, multiple percutaneous coronary interventions or coronary artery bypass grafting that places anastomotic vessels, LAD, or RCA at risk, idiopathic pulmonary fibrosis, advanced stage chronic obstructive pulmonary disease or emphysema), and those who cannot tolerate DIBH. Therefore, with larger patient numbers, we may be able to better define proton versus photon differences for bilateral breast-only treatment in the future; however, we have decided to include such patients in this current study.

Fourth, this study only compared proton to photon treatment using IMRT. It is not clear that IMRT always represents the best alternative for these patients. In particular, DIBH with 3D planning when IMNs are not targeted (or for breast-only cases) may represent a better approach for OAR sparing in comparison with proton therapy. Moreover, the IMRT comparisons did not include DIBH for planning, as the scans selected were the free-breathing ones used for proton therapy treatment. As such, these results may overstate the proton therapy benefits compared with photon since DIBH would have been anticipated to lower heart and lung exposure with IMRT comparisons. However, notwithstanding this limitation, photon studies have shown that it is possible to achieve a mean heart dose reduction of 55.9% (B/CWRT + RNI) and 29.2% (WBRT) with DIBH for left breast irradiation compared with free breathing [40]. Other left breast studies have shown between 25% and 67% mean heart dose reduction with DIBH [41], and recent bilateral breast photon studies show a 22% to 49% reduction with DIBH compared with free breathing [42, 43]. Therefore, while this study only used free breathing, the mean heart dose reduction with protons of 67.8% to 99.7% (reduction, 4.7 Gy-7.2 Gy) is beyond that anticipated using DIBH (22%-55.9%) and highlights that proton therapy likely still offers the proposed material benefits. Fifth, there may be future paradigm shifts in treatment where whole breast radiation, for example, continues to be substituted for approaches such as accelerated partial breast irradiation (aPBI) that minimize the treatment volume needed and thereby lessen the potential benefits of proton seen in current reports for the expansive volumes deployed in many bilateral cases.

Last, the older proton plans did not use the most advanced currently available PBS proton techniques. Preferential avoidance of other structures, such as the articular cartilage of the humeral joints and thyroid, which were not included in the proton optimization algorithm, may be achieved through further dosimetric optimization. In addition, further advantages may be demonstrated when only considering PBS-optimized techniques. However, despite these limitations, the substantial dose reductions to multiple OARs while maintaining target coverage make proton therapy the preferred modality for bilateral breast cancer treatment.
